# Proteomic analysis of the fish pathogen *Flavobacterium columnare*

**DOI:** 10.1186/1477-5956-8-26

**Published:** 2010-06-04

**Authors:** Pradeep R Dumpala, Nagihan Gülsoy, Mark L Lawrence, Attila Karsi

**Affiliations:** 1 Department of Basic Sciences, College of Veterinary Medicine, Mississippi State University, Mississippi State, MS 39762-6100, USA; 2 Department of Biology, Faculty of Art and Science, Marmara University, Göztepe, İstanbul 34722, Turkey

## Abstract

**Background:**

*Flavobacterium columnare *causes columnaris disease in cultured and wild fish populations worldwide. Columnaris is the second most prevalent bacterial disease of commercial channel catfish industry in the United States. Despite its economic importance, little is known about the expressed proteins and virulence mechanisms of *F. columnare*. Here, we report the first high throughput proteomic analysis of *F. columnare *using 2-D LC ESI MS/MS and 2-DE MALDI TOF/TOF MS.

**Results:**

Proteins identified in this study and predicted from the draft *F. columnare *genome were clustered into functional groups using clusters of orthologous groups (COGs), and their subcellular locations were predicted. Possible functional relations among the identified proteins were determined using pathway analysis. The total number of unique *F. columnare *proteins identified using both 2-D LC and 2-DE approaches was 621, of which 10.95% (68) were identified by both methods, while 77.29% (480) and 11.76% (73) were unique in 2-D LC and 2-DE, respectively. COG groupings and subcellular localizations were similar between our data set and proteins predicted from the whole genome. Twenty eight pathways were significantly represented in our dataset (*P *< 0.05).

**Conclusion:**

Results from this study provide experimental evidence for many proteins that were predicted from the *F. columnare *genome annotation, and they should accelerate functional and comparative studies aimed at understanding virulence mechanisms of this important pathogen.

## Background

*Flavobacterium columnare *is a long Gram-negative rod in the family *Flavobacteriaceae*, one of the main phyletic lines within the *Bacteroidetes *group from the domain Bacteria [1]. Several species in *Flavobacteriaceae *cause disease in fish. *F. columnare *is the causative agent of columnaris disease [2], which exists both in fresh and brackish water throughout the world [3]. Outbreaks may result in high mortality, especially during spring and autumn, and are most likely associated with poor environmental conditions causing stress [4,5]. Stressful conditions are common in commercial aquaculture where production is kept at maximum levels.

Columnaris disease generally begins as an external infection on the skin, fins, gills, or oral cavity [6]. On the skin and fins, lesions are characterized by dull, grayish-white or yellow erosive lesions that can progress to deep ulcers in the underlying muscle. External infection often is concurrent with systemic infection and subacute mortalities [3]. In some cases, systemic infection with little or no visible external or internal pathological signs may occur [7]. *F. columnare *infections can be chronic, but more often, the disease appears suddenly and causes mortalities within a few days [6].

A substantial amount of work has been done on *F. columnare *phylogeny [1,8], isolation, identification, and detection of *F. columnare *[9-18], and characterization of *F. columnare *strains [19-25]. In addition, genetic tools for the manipulation of *F. columnare *have recently been reported [26]. Efforts have been made to understand the mechanisms of virulence employed by the organism [27-30], but much remains poorly understood. *F. columnare *produces several extracellular proteases that are believed to be important virulence factors contributing to the branchial and cutaneous necrosis [28,31-33], but the role of the proteases has not been definitively elucidated. A surface capsular material that may be involved in adhesion has been described [34]. Lipopolysaccharide (LPS) may also play an important role in columnaris pathogenesis [35]. Chondroitin lyase activity was found to be significantly related to strain virulence in a temperature dependent manner [30].

Although, 14 *F. columnare *outer membrane related proteins were reported recently [36], little is known about the expressed *F. columnare *proteins. In the present study, we report the first protein expression analysis from *F. columnare *using the complementary technologies of 2-D LC ESI MS/MS and 2-DE MALDI TOF/TOF MS. Results of this study provide experimental evidence for the predicted *F. columnare *proteins and should accelerate functional and comparative studies to delineate pathogenic mechanisms of *F. columnare*.

## Results

### Protein identification using 2-D LC and 2-DE

**Figure 1:**
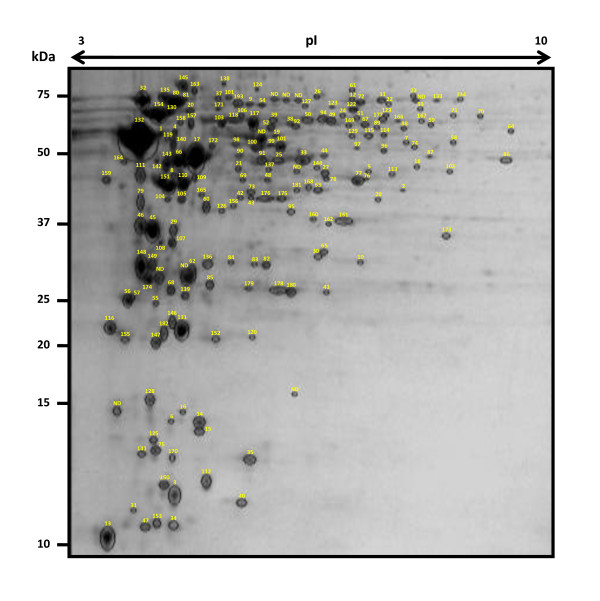
**2-D map of proteins extracted from *Flavobacterium columnare***. Circled spots were excised and analyzed by MS. Numbers indicate proteins with known IDs while NDs indicate unidentified proteins.

2-D LC analysis yielded total 548 *F. columnare *proteins (Additional File 1: Table S1) representing 19.01% of the predicted *F. columnare *proteome (2,882 ORFs). 2-DE analysis of four CBB stained gels showed approximately 600 spots. Among the common spots, 192 were excised and analyzed from one of these gels. We were able to identify 182 (94.79%) of the excised spots (Additional File 2: Table S2), which represented 141 unique *F. columnare *proteins. In 2-DE analysis, 41 (22.53%) proteins were identified in multiple spots, probably due to post-translational modifications and processing. Together, 2-D LC and 2-DE analyses resulted in the identification of 621 *F. columnare *proteins, 480 (77.29%) of which were identified only by 2-D LC, while 73 (11.76%) were identified only by 2-DE (Figure 1), and 68 (10.95%) were detected by both approaches. Analysis of the functional role categories assigned by the J. Craig Venter Institute's Annotation Engine indicated some important categories under cell envelope and cellular processes. Sub categories under these main groups had potential to include proteins that may be involved in *F. columnare *pathogenesis (Table 1).

**Table 1:** *Flavobacterium columnare *potential pathogenesis-related proteins.

ORF #	Protein name	**Approach/SN**^**a)**^	**Role category**^**b)**^
ORF00560	3-deoxy-D-manno-octulosonate 8-phosphate phosphatase, YrbI family	2-D LC	Cell envelope (A)
ORF01244	3-deoxy-D-manno-octulosonate cytidylyltransferase	2-D LC	Cell envelope (A)
ORF02664	Amidinotransferase family protein	2-D LC	Cellular processes (E)
ORF01484	CHU large protein; gliding motility-related protein; putative adhesin AidA-related	2-D LC	Cellular processes (C)
ORF01824	DTDP-4-dehydrorhamnose 3,5-epimerase	2-DE/120	Cell envelope (A)
ORF00198	Extracellular elastinolytic metalloproteinase	2-D LC	Cellular processes (D)
ORF01481	Fibronectin type III domain protein	2-D LC	Cellular processes (D)
ORF02336	GldL	Both/152	Cellular processes (C)
ORF02335	GldM	2-D LC	Cellular processes (C)
ORF02334	GldN	2-DE/151	Cellular processes (C)
ORF02678	Glucose-1-phosphate thymidylyltransferase	2-D LC	Cell envelope (A)
ORF00396	Glycosyl transferase, group 1	2-D LC	Cell envelope (A)
ORF02332	Glycosyltransferase, putative	2-D LC	Cell envelope (A)
ORF00532	GTP-binding protein TypA/BipA	2-DE/36, 37	Cellular processes (F)
ORF01035	Hemagglutinin	2-D LC	Cellular processes (E)
ORF02801	Lipid-A-disaccharide synthase	2-D LC	Cell envelope (A)
ORF01136	LPS biosynthesis protein , RfbU family	2-D LC	Cell envelope (A)
ORF02060	Mannosyltransferase	2-D LC	Cell envelope (A)
ORF00475	Membrane protein, putative	2-D LC	Cellular processes (E)
ORF01989	Monofunctional biosynthetic peptidoglycan transglycosylase	2-D LC	Cell envelope (B)
ORF02568	PepSY-associated TM helix family	2-D LC	Cellular processes (C)
ORF02672	Polysaccharide export outer membrane protein	2-D LC	Cell envelope (A)
ORF01163	Tetraacyldisaccharide 4'-kinase	2-D LC	Cell envelope (A)
ORF01370	UDP-3-0-acyl N-acetylglucosamine deacetylase	2-D LC	Cell envelope (A)
ORF01367	UDP-3-O-[3-hydroxymyristoyl] glucosamine N-acyltransferase	2-D LC	Cell envelope (A)
ORF02676	UDP-glucose 6-dehydrogenase	Both/169	Cell envelope (A)
ORF02536	UDP-N-acetylglucosamine 2-epimerase	2-D LC	Cell envelope (A)
ORF00211	Universal stress protein family protein	2-DE/10	Cellular processes (F)

a) SN indicates spot number marked on the 2-DE image (Figure 1).

b) A, Biosynthesis and degradation of surface polysaccharides and lipopolysaccharides; B, Biosynthesis and degradation of murein sacculus and peptidoglycan; C, Chemotaxis and motility; D, Pathogenesis; E, Toxin production and resistance; F, Adaptations to atypical conditions.

### Functional classification of identified proteins

**Figure 2:**
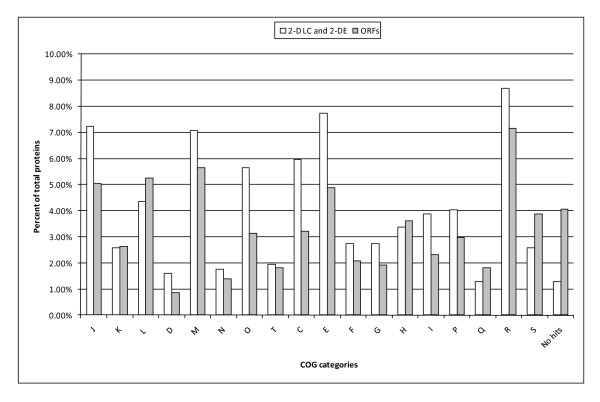
**Comparison of COG categories**. Proteins identified in this study (621 proteins in both 2-D LC and 2-DE analysis) and predicted from the *Flavobacterium columnare *draft genome (2,882 ORFs) were organized into COG functional groups. Percentages were calculated by dividing the number of proteins in the particular COG category by the number of unique proteins in each analysis. COG categories are as follows: J, translation, ribosomal structure and biogenesis; K, transcription; L, replication, recombination, and repair; D, cell cycle control, cell division, chromosome partitioning; M, cell wall/membrane/envelop biogenesis; N, cell motility; O, post-translational modification and protein turnover, chaperones; T, signal transduction mechanisms; U, intracellular trafficking, secretion, and vesicular transport; V, defense mechanisms; C, energy production and conversion; E, amino acid transport and metabolism; F, nucleotide transport and metabolism; G, carbohydrate transport and metabolism; H, coenzyme transport and metabolism; I, lipid transport and metabolism; P, inorganic ion transport and metabolism; Q, secondary metabolites biosynthesis, transport and catabolism; R, general function prediction only; S, function unknown. "No hit" group indicates non-significant short or low-complexity sequences. Query proteins not belong to any of the currently-defined COGs (23.51% in 2-D LC and 2-DE analysis and 36.33% in predicted ORFs) were not included in the figure for clarity.

Functional classifications of the 621 identified unique proteins and the 2,882 predicted *F. columnare *proteins are summarized in Figure 2. A total of 88 (14.17%, 88/621) proteins from our dataset and 372 (12.91%, 372/2,882) proteins from the predicted whole proteome were classified as information storage and processing related categories (J, K, and L). Cellular processes and signaling related categories (D, M, N, O, and T) included 112 (18.04%) and 370 (12.84%) proteins from our protein dataset and the whole proteome database, respectively. A total of 197 (31.72%) and 658 (22.83%) proteins from our protein dataset and the predicted whole proteome, respectively, were represented in metabolism related categories (C, E, F, G, H, I, P, and Q). Poorly characterized COG groups (R and S) contained 70 (11.27%) and 318 (11.03%) proteins in our protein dataset and the predicted whole proteome, respectively. "No COGs" (proteins not belong to any of the currently-defined COGs) was the top represented category in our protein dataset (23.51%) and in the whole proteome (36.33%). Eight (1.29%) proteins from our protein dataset and 117 (4.06%) proteins from whole proteome were assigned to "No hit" (non-significant short or low-complexity sequences) category. A summary of Pfam domain analysis on proteins detected by 2-D LC and 2-DE, and predicted from the *F. columnare *draft genome are given in Additional Files 3, 4, and 5: Tables S3, S4 and S5, respectively.

### Subcellular localization of identified proteins

**Figure 3:**
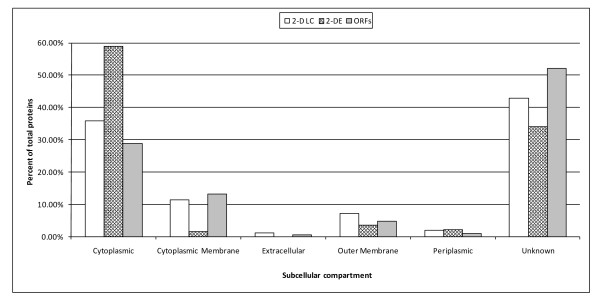
**Subcellular locations of *Flavobacterium columnare *proteins determined by PSORTb prediction**. Percentages were calculated by dividing the number of proteins in the particular subcellular location by the number of unique proteins in each analysis (548 proteins in 2D-LC, 141 proteins in 2-DE, and 2,882 ORFs predicted from the draft genome). 2-D LC and 2-DE indicates proteins identified in this study, while ORFs indicates proteins predicted from the whole genome. Unknown category includes proteins with multiple subcellular locations or unknown location.

Subcellular locations of *F. columnare *proteins identified in this study and predicted from the draft genome were determined (Figure 3). 2-DE and 2-D LC generally resulted in similar coverage of subcellular compartments compared to the predicted proteome, except 2-DE gave better coverage of cytoplasmic proteins and poorer coverage of cytoplasmic membrane proteins. 2-D LC had better representation of proteins from both membrane compartments and extracellular proteins. Both 2-D LC and 2-DE had lower representation of the "unknown" subcellular location proteins than the percentage from the predicted proteome. In this study, 42 proteins were predicted to locate in the *F. columnare *outer membrane by PSORTb (Table 2).

**Table 2:** Outer membrane proteins of *Flavobacterium columnare *predicted by PSORTb.

Protein name	ORF #	**COG**^**a)**^	**Approach/SN**^**b)**^
Conserved hypothetical protein	ORF02881	-	2-D LC
Conserved hypothetical protein	ORF00226	-	2-D LC
Conserved hypothetical protein	ORF01938	D	2-D LC
Conserved hypothetical protein	ORF00224	-	2-D LC
Conserved hypothetical protein	ORF02143	-	2-D LC
Conserved hypothetical protein	ORF02328	R	2-D LC
Extracellular elastinolytic metalloproteinase	ORF00198	-	2-D LC
Fjo24	ORF02628	-	2-D LC
Hypothetical protein	ORF02366	-	2-D LC
Hypothetical protein	ORF01273	-	2-D LC
Hypothetical protein	ORF01272	-	2-D LC
Hypothetical protein	ORF01485	-	2-D LC
Hypothetical protein	ORF02042	P	2-D LC
Lipoprotein, putative	ORF01143	-	2-D LC
Lipoprotein, putative	ORF02619	-	Both/166, 167
Omp assembly complex, YaeT protein	ORF02858	M	2-D LC
OmpA	ORF02005	M	Both/132
OmpA	ORF00341	M	2-D LC
OmpA family protein	ORF00085	M	2-D LC
OmpA/MotB family	ORF00937	M	2-D LC
OmpA/MotB family	ORF01639	N	2-D LC
OmpA/MotB family	ORF00225	M	2-DE/11, 12
Outer membrane efflux protein	ORF00073	MN	2-D LC
Outer membrane insertion C- signal domain protein	ORF00187	-	2-D LC
Peptidyl-prolyl cis-trans isomerase C	ORF02829	O	2-D LC
Putative TonB-dependent receptor	ORF00266	P	2-D LC
RagA protein, putative	ORF00258	P	2-D LC
Secreted peptidase, family M23	ORF01434	M	2-D LC
Secreted protein	ORF02176	-	2-DE/141
Snf2 family helicase	ORF00742	KL	2-D LC
Tetratricopeptide repeat domain protein	ORF01855	R	2-D LC
TonB-dependent outer membrane receptor	ORF00779	P	2-D LC
TonB-dependent outer membrane receptor	ORF02179	P	2-D LC
TonB-dependent outer membrane receptor	ORF00147	P	2-D LC
TonB-dependent outer membrane receptor	ORF02172	P	2-D LC
TonB-dependent outer membrane receptor	ORF02232	P	2-D LC
TonB-dependent outer membrane receptor	ORF01690	P	2-D LC
TonB-dependent outer membrane receptor, putative	ORF00704	P	2-D LC
TonB-dependent outer membrane receptor, putative	ORF02424	P	2-D LC
TonB-dependent receptor plug domain protein	ORF01433	P	2-D LC
TonB-dependent receptor, putative	ORF01511	P	2-D LC
Transcriptional regulator, AraC family protein	ORF01879	R	2-D LC

a) COG categories are as follows: M, cell wall/membrane/envelop biogenesis; D, cell cycle control, cell division, chromosome partitioning; P, inorganic ion transport and metabolism; S, function unknown; N, cell motility; R, general function prediction only; O, post-translational modification and protein turnover, chaperones; K, transcription; L, replication, recombination and repair; -, "No COGs".

b) SN indicates spot number marked on the 2-DE image (Figure 1).

### Pathway analysis

**Figure 4:**
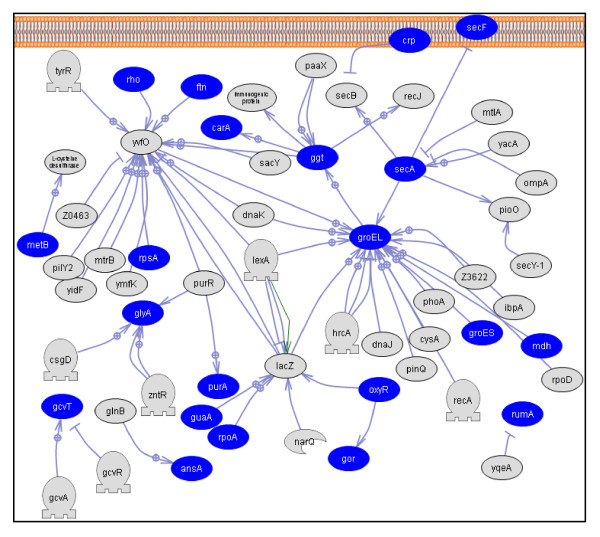
**Amino acid metabolism related pathways in *Flavobacterium columnare***. Amino acid metabolism related pathways include glutamate; histidine; selenoamino acid; alanine and aspartate; glycine, serine, and threonine metabolism; lysine biosynthesis; lysine degradation; and valine, leucine, and isoleucine degradation pathways. Entities shown in oval represent genes involved in this pathway. Filled ovals represent proteins detected in this study. Unconnected genes were removed from the figure to reduce complexity.

We used Pathway Studio analysis to gain insight into various pathways represented in *F. columnare *proteins identified by 2-D LC and 2-DE analysis. Twenty seven pathways belonging to metabolism and one pathway in genetic information processing were significantly represented (*P *< 0.05, Additional File 6: Table S6). Out of 28 pathways, eight related to amino acid metabolism (Figure 4), five carbohydrate metabolism, four cofactor and vitamin metabolism, four xenobiotics biodegradation and metabolism, two glycan biosynthesis and metabolism, two nucleotide metabolism, one energy metabolism, one biosynthesis of secondary metabolites, and one related to translational process. 277 proteins from our dataset were integral to these pathways. Of the significantly represented pathways, purine metabolism contained the highest number of proteins (24 proteins), while biotin metabolism pathway had the fewest (4 proteins). Of all the pathways, folate biosynthesis pathway was the most significant pathway in our dataset (*P *= 2.25E-09), which was followed by purine metabolism pathway (*P *= 1.44E-07).

## Discussion

This research analyzed the soluble cell proteome of *Flavobacterium columnare *using complementary technologies of 2-D LC and 2-DE. Proteomic data from this study has provided experimental evidence for the expression of *F. columnare *proteins that were previously only predicted from the draft genome sequence. Data from this study provides a picture of the expressed *F. columnare *proteome during log-phase growth in FCGM along with a 2-D reference map, which will provide an important basis for comparison under other environmental conditions.

Currently, the most popular methods employed to explore protein expression utilize protein separation by 2-D LC (gel-free) or 2-DE (gel-based) followed by MS analysis [37]. Use of more than one methodology in proteomic investigations improves proteome coverage because each method identifies a set of unique proteins [38,39]. Therefore, in the present study, we employed the complementary techniques of 2-DE and 2-D LC to identify 621 unique proteins from *F. columnare *(480 unique to 2-D LC, 73 unique to 2-DE, and 68 common to both), which provided experimental evidence for more than one fifth (21.55%) of the predicted 2,882 *F. columnare *ORFs.

Ribosomal proteins, one of the most abundantly expressed protein types in cells, were detected in high numbers. In the draft *F. columnare *genome sequence, 60 proteins were predicted as ribosomal proteins. In the present study, we identified 14 and 3 unique ribosomal proteins using 2-D LC and 2-DE, respectively. Along with ribosomal proteins, we also identified several proteins involved in translational machinery, including several aminoacyl-tRNA synthases, translation initiation factors such as translation initiation factor IF-2 (InfB) (ORF00214), and elongation factors (EF) such as transcription elongation factor GreA (ORF00178), translation elongation factor Tu (ORF00244), translation elongation factor Ts (ORF00695), translation elongation factor P (ORF01368), and translation elongation factor G (ORF02228).

Outer membrane proteins (OMPs) often act as important virulence factors in Gram-negative bacteria. Therefore, identification of such molecules provides targets for future studies to increase our understanding of *F. columnare *pathogenesis. Moreover, OMPs may also play a crucial role in interacting with other molecules. In the present study, we identified 42 unique OMPs using 2-D LC and 2-DE analysis, which includes seven of the 14 OMPs previously identified by Liu et al. (p7, p8, p9, p10, p11, p12, p16) [36]. TonB-dependent outer membrane receptors function in the import of iron-siderophore complexes and vitamin B12 across the outer membrane in Gram-negative bacteria [40,41]. We identified six OmpA related proteins, which are the most abundant protein components of outer membranes of Gram-negative bacteria [42]. OmpA is predicted to be a nonspecific diffusion channel, allowing small solutes to cross the outer membrane [43].

In *F. columnare*, proteases, adherence factors, and chondroitin AC lyase have been reported as virulence factors [28,44]. The *F. columnare *genome encodes eight putative secreted metalloproteases/peptidases probably involved in virulence and/or in the destruction of host tissues. In this study, we identified only one extracellular elastinolytic metalloproteinase (ORF00198) using 2-D LC, which is probably due to the absence of host/host factors during *F. columnare *growth and/or isolation of the bacterial cell proteins, but not the secreted proteins. Chondroitin AC lyase was not detected, but we did detect fibronectin type III domain protein (ORF01481), a potential adherence factor that may be involved in cell surface binding of *F. columnare*. LemA (ORF00319) is also shown to be a putative virulence factor in several animal and plant bacteria especially in *Pseudomonas *[45,46].

*F. columnare *moves over surfaces by gliding motility. Thirteen gliding motility related proteins are encoded in the *F. columnare *draft genome, out of which we detected four (GldN, GldM, GldL and gliding motility-related protein). Detection of only four gliding motility proteins may be due to planktonic growth of *F. columnare *in broth culture.

Production of reactive oxygen species (ROS) by macrophages is one of the most effective defense mechanisms of host against bacterial pathogens [47]. Superoxide dismutases, bifunctional catalase-peroxidases, thiol peroxidases, and proteins of the peroxy-redoxin family are important bacterial defense mechanisms allowing survival of *F. psychrophilum *against phagocytes [48-52]. In the present study, we identified copper/zinc superoxide dismutase (SodC) (ORF02131), superoxide dismutase [Mn] (ORF02002), catalase/peroxidase HPI (KatG) (ORF02145), cytochrome c551 peroxidase (ORF01733), thiol peroxidase (ORF00815), and glutathione peroxidase (ORF00555). SodC is a periplasmic enzyme that converts superoxide radicals to hydrogen peroxide and water. Sod [Mn] is expressed only under aerobic conditions [53] and is believed to be involved in effective prevention of DNA damage in *Escherichia coli *[54]. Expression of these proteins in *F. columnare *suggests its ability to resist ROS.

The predicted *F. columnare *proteins were classified into COGs to assign functions. The majority of proteins identified in the current study (31.72%) were housekeeping proteins in "metabolism" related categories ('metabolism', 'cellular processes and signaling', and 'information storage and processing'). This was not surprising because we harvested proteins from bacteria when they were metabolically active at mid log phase. As compared to the predicted whole proteome, our experimental dataset had relatively lower coverage of the *F. columnare *proteins that were not assigned to COG groups. Proteins with unknown function may be expressed only under specialized conditions like host environment or stressed conditions and may not be expressed under laboratory culture conditions. Analysis of metabolism-related proteins in our list implies that amino acids, instead of carbohydrates, could be the major sources of carbon or energy. The FCGM medium we used for bacterial growth is a good source of amino acids, nitrogen, and certain salts like MgSO_4 _and CaCl_2 _but it lacks readily available carbohydrate sources. Most of the metabolic proteins indentified in our study were amino acid kinases, including five histidine kinases and five aminotransferases. Only three carbohydrate kinases involved in core carbohydrate metabolisms were identified. We found 18 peptidases, which may be useful in utilizing available amino acids as a major source of carbon. Previous studies on *F. psychrophilum *also suggest that this bacterium was unable to use readily available carbohydrates as a major energy source [55].

We used the PSORTb algorithm to predict the subcellular locations of *F. columnare *proteins. Many proteins were mapped to the cytoplasm, which is expected because these proteins are not hydrophobic, and thus solubility does not hinder protein isolation and separation. 2-DE was particularly effective at detection of cytoplasmic proteins. By contrast, cytoplasmic and outer membrane proteins were identified in higher percentages using 2-D LC when compared to 2-DE. The same buffer solution containing 7M urea and 2% CHAPSO was used to solubilize proteins during isolations for both 2D-LC and 2-DE, so it appears that the difference is not due to the isolation procedure, but rather the separation techniques. Other studies have reported that hydrophobic membrane proteins are typically difficult to identify using 2-DE. Hydrophobic proteins were found to be under-represented in 2-DE based proteome analyses of membrane protein fractions from *E. coli *[56-58] and *Bacillus subtilis *[39,59,60]. It is important to keep in mind that we chose to analyze only 192 common spots (30.81%) out of approximately 600 spots identified on our gels, so the bias toward cytoplasmic proteins in our 2-DE results may be because these proteins were more abundant or consistently present in gels. However, for future functional studies of membrane proteins, specialized isolation techniques may be needed. The ease and higher sensitivity of 2-D LC for detection of hydrophobic membrane proteins may make this the preferred method for studying membrane proteins.

From the Pathway Studio analysis, we identified pathways that were significantly represented in our protein dataset. Folate biosynthesis was the most significantly represented metabolic pathway, which was followed by purine metabolism pathway. Higher representation of metabolic and translational process related pathways is expected because proteins were isolated from *F. columnare *during a metabolically active state. Amino acid metabolism related pathways (glutamate; histidine; selenoamino acid; alanine and aspartate; glycine, serine, and threonine metabolism; lysine biosynthesis; lysine degradation; and valine, leucine, and isoleucine degradation) are highly represented in our dataset compared to carbohydrate metabolism. Moreover, we have identified 18 peptidases in our protein list suggesting breakdown of host proteins and usage of resultant amino acids as a major source of energy, carbon, and nitrogen by *F. columnare*. Similarly, Glycosylphosphatidylinositol (GPI)-anchor biosynthesis and LPS biosynthesis pathways were significantly represented in our dataset, which may play an important role in cell wall synthesis. Moreover, LPS biosynthesis may be one of the important virulence-related systems in *F. columnare *pathogenesis. LPS is an important component of the cell envelope in Gram-negative bacteria and is an immunodominant antigen.

## Conclusions

Our main objective was to identify the expressed proteins of *F. columnare *during normal growth. We showed for the first time the expression of 621 proteins from *F. columnare*, which provides experimental evidence for many proteins that were predicted from the *F. columnare *genome annotation. We expect this information could accelerate functional and comparative studies aimed at understanding virulence mechanisms of this important pathogen. For example, comparative protein expression analysis between low and high virulence *F. columnare *strains could help determine proteins involved in virulence. Furthermore, orthologous protein mapping of identified *F. columnare *proteins to well studied bacteria would reveal protein targets for mutational analysis to understand gene function as well as to develop live attenuated vaccines.

## Methods

### Protein extraction

*F. columnare *growth medium (FCGM) [tryptone (8.00 g), yeast extract (0.80 g), MgSO_4 _7 H_2_O (1.00 g), CaCl_2 _2 H_2_O (0.74 g), NaCl (5.00 g), and sodium citrate (1.50 g) per liter] was used to grow *F. columnare*. Isolate ATCC 49512 was streaked on FCGM agar plates (FCGM medium plus 8.00 g agar per liter) and incubated at 30°C for 48 h. Four colonies were grown in 12 ml FCGM broth separately at 30°C under continuous shaking at 200 rpm. Growth of bacteria was monitored by measuring optical density at 600 nm (OD_600_), and bacteria were harvested at mid-exponential phase (OD_600 _0.6) by centrifugation at 3,750 rpm for 15 min at 30°C.

The four bacterial pellets were separately resuspended in cold urea-CHAPS buffer (7 M urea, 50 mM tris-HCl, 2% CHAPS, 8 mM PMSF pH 8.0), and cells were lysed immediately on ice by applying ten intermittent pulses of 10 s with a sonicator. Bacterial homogenates were centrifuged at 14,000 rpm for 5 min at 4°C to remove cell debris and unbroken cells. Proteins from supernatant were precipitated by trichloroacetic acid/Acetone, and the resultant protein pellets were resuspended in rehydration buffer (7 M urea, 20 mM tris-HCl, 5 mM EDTA, 5 mM MgCl_2_, 4% CHAPS, and 5 mM PMSF pH 8.0). Protein concentrations were estimated using a 2-D Quant Kit (GE Healthcare, Piscataway, NJ).

### 2-D LC ESI MS/MS analysis

100 μg of protein was digested with trypsin (1:50 w/w) at 37°C for 16 h. The resultant peptides were desalted, dried in a vacuum centrifuge, and resuspended in 20 μL of 5% acetonitrile and 0.1% formic acid. Mass spectrometric analysis was accomplished using a ProteomeX Workstation (Thermo Scientific, Waltham, MA) as described previously [61]. The *F. columnare *draft genome was annotated using Annotation Engine at the J. Craig Venter Institute, which uses the Glimmer algorithm for gene prediction [62,63]. The *F. columnare *protein database with 2,882 open reading frames (ORFs) is available on our website http://www.miangel.msstate.edu, while the *F. columnare *genome project is hosted at The Oklahoma University Health Sciences Center http://microgen.ouhsc.edu. To identify proteins, mass spectra and tandem mass spectra were searched against the *in silico *trypsin digested *F. columnare *protein database as previously reported [64]. Protein identifications and associated MS data were submitted to the PRIDE database [Accession number: 9749].

### 2-DE and MALDI MS analysis

Proteins were extracted from four mid-exponential phase bacterial cultures as described in section 2.1. Protein pellets were resuspended in 350 μL of freshly prepared rehydration buffer (7 M urea, 2 M thio urea, 2% CHAPS, 1:50 carrier ampholytes, 0.3% DTT) and quantified using 2-D Quant Kit (GE Healthcare). For in-gel rehydration, 800-1000 μg of solubilized proteins were loaded onto each IPG strip (17 cm pH 3-10 NL). IEF, in-gel trypsin digestion, and MALDI MS analysis were performed as previously described [64,65].

### Identification of COGs, domains, protein locations, and pathways

All identified proteins from our dataset and the predicted whole *F. columnare *proteome were organized into COG functional groups using COGnitor tool [66-68]. Similarly, protein domain analysis was conducted by using batch Pfam analysis [69]. The subcellular location of proteins from our dataset as well as from the whole *F. columnare *proteome were predicted using PSORTb v2.0.4 [70,71]. Pathways with significant protein representation in our experimentally derived protein dataset were identified using Pathway Studio (Ariadne Genomics, Rockville, MD). Due to lack of a molecular interaction database for *F. columnare *in Pathway Studio, all *F. columnare *proteins were mapped to their orthologs in *E. coli*, *F. psychrophilum*, and *F. johnsoniae *by identifying reciprocal-best-BLAST hits. The resulting ortholog map file was used to predict pathways in *F. columnare *proteins. We used *P *≤ 0.05 to select pathways with significant protein coverage.

## Competing interests

The authors declare that they have no competing interests.

## Authors' contributions

PRD carried out the experiments, analysis and interpretation of the data, and preparation of the manuscript. NG participated in the experiments and preparation of the manuscript. MLL contributed to preparation and critical review of the manuscript. AK contributed to the overall conception and design of the project as well as preparation and critical review of the manuscript. All authors read and approved the final manuscript.

## Supplementary Material

Additional file 1 **Table S1 - *Flavobacterium columnare *proteins identified by 2-D LC analysis**. Click here for file

Additional file 2 **Table S2 - *Flavobacterium columnare *proteins identified by 2-DE analysis**. Click here for file

Additional file 3 **Table S3 - Pfam analysis of proteins identified by 2-D LC analysis**. Click here for file

Additional file 4 **Table S4 - Pfam analysis of proteins identified by 2-DE analysis**. Click here for file

Additional file 5 **Table S5 - Pfam analysis of proteins predicted from the *Flavobacterium columnare *genome**. Click here for file

Additional file 6 **Table S6 - Pathways significantly represented in *Flavobacterium columnare *protein dataset**. Click here for file
