# Supplementary material for: Proteomic analysis of the fish pathogen Flavobacterium columnare
Source: Proteome Sci. 2010 Jun 4;8:26. doi: 10.1186/1477-5956-8-26 (PMC2890538; doi:10.1186/1477-5956-8-26)
Supplement: Additional file 6 — Table S6 - Pathways significantly represented in Flavobacterium columnare protein dataset. [file 1477-5956-8-26-S6.DOC]

**Table S3: Pathways significantly represented in *Flavobacterium columnare* protein dataset.**

| **Pathway name** | **No. of proteins** | **Total no. of entities** | ***P* value** | **Classification** |
| --- | --- | --- | --- | --- |
| Folate biosynthesis | 20 | 162 | 2.25E-9 | Metabolism of Cofactors and Vitamins |
| Purine metabolism | 24 | 289 | 1.44E-7 | Nucleotide Metabolism |
| Benzoate degradation via CoA ligation | 14 | 156 | 2.98E-5 | Xenobiotics Biodegradation and Metabolism |
| Citrate cycle (TCA cycle) | 10 | 82 | 3.19E-5 | Carbohydrate Metabolism |
| Aminoacyl-tRNA synthetases | 10 | 83 | 3.54E-5 | Translation |
| Pyrimidine metabolism | 15 | 204 | 1.55E-4 | Nucleotide Metabolism |
| Oxidative phosphorylation | 10 | 100 | 1.76E-4 | Energy Metabolism |
| Ethylbenzene degradation | 8 | 75 | 5.12E-4 | Xenobiotics Biodegradation and Metabolism |
| Propanoate metabolism | 11 | 136 | 5.42E-4 | Carbohydrate Metabolism |
| Valine, leucine and isoleucine degradation | 10 | 116 | 5.89E-4 | Amino Acid Metabolism |
| Pyruvate metabolism | 12 | 163 | 7.13E-4 | Carbohydrate Metabolism |
| Glutamate metabolism | 9 | 102 | 9.38E-4 | Amino Acid Metabolism |
| Alanine and aspartate metabolism | 9 | 104 | 1.08E-3 | Amino Acid Metabolism |
| Selenoamino acid metabolism | 9 | 105 | 1.15E-3 | Metabolism of Other Amino Acids |
| Limonene and pinene degradation | 11 | 150 | 1.23E-3 | Biosynthesis of Secondary Metabolites |
| Glycolysis / Gluconeogenesis | 11 | 151 | 1.30E-3 | Carbohydrate Metabolism |
| Glycine, serine and threonine metabolism | 12 | 191 | 2.79E-3 | Amino Acid Metabolism |
| Glycosylphosphatidylinositol(GPI)-anchor biosynthesis | 5 | 41 | 3.29E-3 | Glycan Biosynthesis and Metabolism |
| 1,1,1-Trichloro-2,2-bis(4-chlorophenyl)ethane (DDT) | 6 | 63 | 4.61E-3 | Xenobiotics Biodegradation and Metabolism |
| Ubiquinone biosynthesis | 8 | 115 | 7.77E-3 | Metabolism of Cofactors and Vitamins |
| Butanoate metabolism | 9 | 172 | 2.76E-2 | Carbohydrate Metabolism |
| One carbon pool by folate | 5 | 72 | 3.33E-2 | Metabolism of Cofactors and Vitamins |
| Caprolactam degradation | 5 | 72 | 3.33E-2 | Xenobiotics Biodegradation and Metabolism |
| Histidine metabolism | 8 | 150 | 3.35E-2 | Amino Acid Metabolism |
| Lipopolysaccharide biosynthesis | 7 | 124 | 3.49E-2 | Glycan Biosynthesis and Metabolism |
| Lysine degradation | 9 | 180 | 3.56E-2 | Amino Acid Metabolism |
| Biotin metabolism | 4 | 53 | 4.25E-2 | Metabolism of Cofactors and Vitamins |
| Lysine biosynthesis | 6 | 104 | 4.50E-2 | Amino Acid Metabolism |
